# Antiquity of Artificial Teeth

**Published:** 1891-02

**Authors:** 


ANTIQUITY OF ARTIFICIAL TEETH.—Dr. Van Marter, of Rome, Italy, has discovered in many of the skulls in different Etruscan tombs, as well as in those deposited in the various museums, interesting specimens of ancient dentistry work and artificial teeth. The false teeth were in most cases carved from those of some large animal, and in many instances were fastened to the natural ones by gold bands. The skulls examined date as far back as six centuries before Christ, which proves that dentistry is not a modern art.

